# P-1098. Clinical outcomes in four patients with refractory Coccidioidal meningitis treated with olorofim

**DOI:** 10.1093/ofid/ofae631.1286

**Published:** 2025-01-29

**Authors:** Michelle Yousefzadeh, David Goodman-Meza, Paul R Allyn

**Affiliations:** UCLA, LOS ANGELES, California; UCLA, LOS ANGELES, California; UCLA, LOS ANGELES, California

## Abstract

**Background:**

Coccidioidal meningitis (CM) remains a highly morbid condition. Olorofim is a novel agent that inhibits the fungal dihydroorotate dehydrogenase enzyme and has potent in vitro activity against Coccidioidomycosis.

**Figure:**
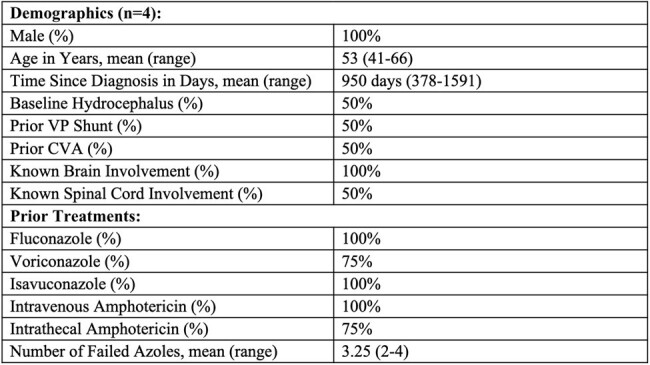

Demographics of patients at the time of olorofim initiation with description of prior treatments.

**Methods:**

We performed a retrospective case series of 4 patients with refractory CM treated with olorofim between 2020 and 2024 comparing outcomes assessed by symptoms, brain imaging, and Mycosis Study Group (MSG) score.

**Figure:**
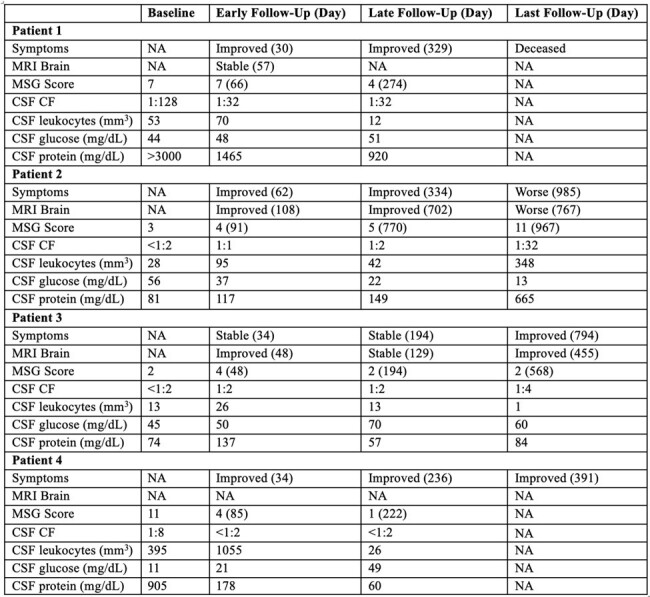

Change in Symptoms, MRI Brain Imaging, MSG Score, and CSF Parameters after Olorofim Initiation. Abbreviations: CF, Coccidiodes complement fixation; CSF, cerebrospinal fluid; MRI, magnetic resonance imaging, MSG, Mycosis Study Group (MSG).

**Results:**

At baseline, mean age was 53 (range 41-66), mean time since diagnosis 950 days (range 378-1591), mean MSG score 5.8 (range 3-11), and mean azoles failed 3.25 (range 2-4). Follow-up occurred at variable intervals for a total duration of 332-985 days on therapy. In early follow-up, symptoms were improved in 3 patients and stable in 1; available imaging was improved in 2 patients and stable in 1; mean MSG score was 4.75, decreased in 1 patient, unchanged in 1, and increased but low in 2. In late follow-up, symptoms were improved in 3 patients and stable in 1; available imaging was improved in 1 patient and stable in 1; mean MSG score was 2.7, decreased in 2 patients, unchanged in 1, and increased in 1. Patient 3 developed significant transaminitis requiring dose reduction at day 91 with subsequent relapse (MSG score 14) but was eventually stabilized on combination therapy with fluconazole.

**Figure:**
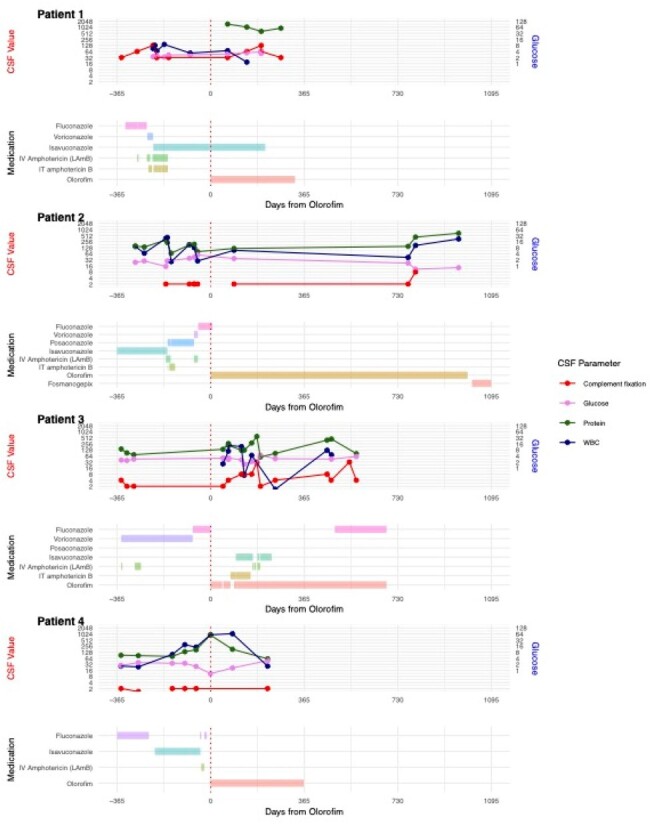

Cerebral spinal fluid (CSF) complement fixation, glucose, protein and white blood cell count (WBC) graphed over time on antifungal treatment. Time 0 represents initiation of olorofim.

**Conclusion:**

At last follow-up, 2 patients were doing well with improvement or stability in all domains, 1 patient died of unrelated causes, and 1 patient had late treatment failure after prolonged stability for 2.5 years. Olorofim may be an effective alternative treatment for patients with refractory CM.

**Figure:**
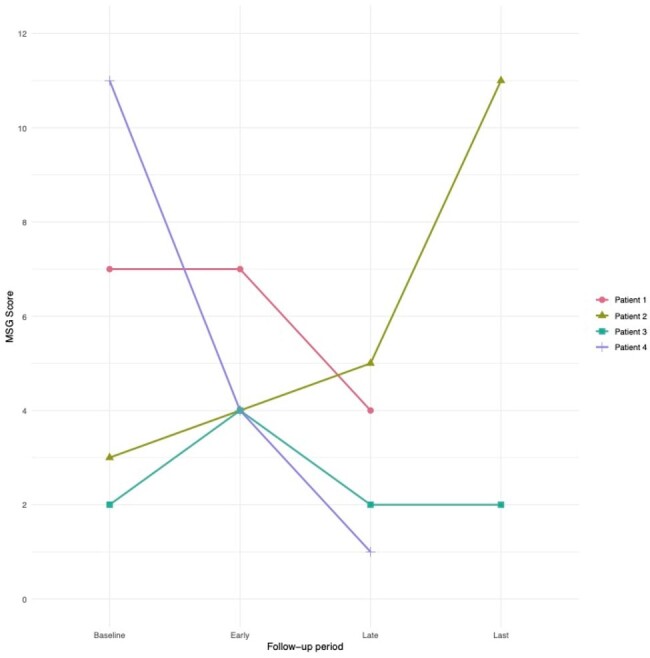

Mycosis Study Group (MSG) scores plotted over time. The plot excludes an MSG score of 14 for patient 3 shortly after Early follow-up period during a temporary hold on olorofim administration due to medication induced transaminitis.

**Disclosures:**

**All Authors**: No reported disclosures

